# Efficacy and safety of acupuncture for urinary retention: A protocol for systematic review and meta analysis

**DOI:** 10.1097/MD.0000000000021511

**Published:** 2020-08-14

**Authors:** Qinyu Zhao, Bao Song, Shenshen Chen, Yongzheng Zhu, Hongling Jia, Shenyang Liu

**Affiliations:** a College of Acupuncture and Tuina, Shandong University of Traditional Chinese Medicine; b Department of Tuina, Affiliated Hospital of Shandong University of Traditional Chinese Medicine; c Department of Hematology, The First Affiliated Hospital of Shandong First Medical University; d Department of Acupuncture, The Second Affiliated Hospital of Shandong University of Traditional Chinese Medicine, Jinan; e Pain Department, Weifang Traditional Chinese Medicine Hospital, Weifang, Shandong, PR China.

**Keywords:** acupuncture, meta-analysis, systematic review, urinary retention

## Abstract

**Background:**

Many postoperative patients and males suffer urinary retention (UR) symptom, which directly affects their quality of life. Acupuncture is widely used by Traditional Chinese Medicine doctors to treat various postoperative urinary retention and elderly male urinary retention patients. However, acupuncture treatment of urinary retention symptoms lacks relevant multi-center clinical studies and lacks a more comprehensive meta-analysis report, which contradicts clinical practice. To confirm the safety and efficacy of acupuncture treatment of urinary retention caused by various reasons requires more comprehensive and strong evidence-based medical evidence.

**Methods:**

Databases including CNKI, PubMed, Web of Science, Cochrane Central Register of Controlled Trials, Scopus, EBSCO were retrieved for relevant literature, with the retrieval deadline being June 23, 2020. The 2 conducted independent reading of the retrieved literature and removed the duplications, and then used the Cochrane Handbook to score the randomized controlled trials (RCTs). Only when the score is greater than 5 points can they be included. Then extract the basic information of the included literature, post-voided residual urine (PVR), maximal cystometric capacity (MCC), maximal flow rate (MFR), bladder compliance (BC), adverse events (AE), and effective rate data, and make a literature feature table. The methodological quality was evaluated with the “Risk of Bias” tool, and the meta-analysis was performed by using the RevMan 5.3.5 software. Use Stata 15 for regression analysis to find the source of heterogeneity, and try to resolve it using subgroup analysis.

**Results:**

The analysis of PVR, MCC, MFR, BC, AE and effective rate data can provide high-quality evidence for high-quality synthesis and/or descriptive analysis of the effectiveness and safety of acupuncture treatment of various causes of urinary retention.

**Conclusion:**

This study can provide more comprehensive evidence to prove whether acupuncture is effective and safe for patients with UR.

**Registration:**

The research has been registered and approved on the PROSPERO website. The registration number is CRD42019119238.

## 1 Introduction

### 1.1 Background introduction

Today, many postoperative patients and males suffer urinary retention (UR) symptom, which directly affects their quality of life. The National Institute of Diabetes and Digestive and Kidney Diseases defined urinary retention as a series of symptoms of bladder incomplete or does not empty at all. Urinary retention can be divided into acute urinary retention (AUR) and chronic urinary retention (CUR) according to the rate of onset. The European Association of Urology (EAU) and the American Urological Association (AUA) classified urinary retention in the lower urinary tract category.^[1,2]^ Nevertheless, the exact definitions of AUR and CUR remain controversial.^[3]^ Urinary retention is a common complication after anaesthesia and surgery, with reported rates ranging from 5% to 70%.^[1]^ That can also be caused by prostate problems.^[3]^ Urinary retention always takes a lot of trouble such as urinary tract infection and may prolong hospital stay and increase discharge time in outpatients.^[4,5]^ The incidence of urinary retention has been shown to increase with age, with a 2.4-fold increased risk of retention in patients over 50 years of age.^[6,7]^ Catheterization is a common way to deal with postoperative urinary retention, but catheterization also is the leading cause to increase risk of urinary tract infections, the incidence of 8%, and hospital mortality.^[8,9]^ Some reports show that Earlier removal of bladder catheter in surgical patients receiving thoracic epidural analgesia can decrease the incidence of urinary tract infection.^[10]^ Acupuncture is an effective method to decrease the incidence of urinary retention, and urinary tract infection and shortens hospital stay.^[11,12]^

### 1.2 Intervention method function introduction

Acupuncture became a hot topic in western countries about 40 years ago and has gained international fame since James Restons piece, “Now, Let Me Tell You about My Appendectomy in Peking,” was published.^[13]^ Many scholars know that acupuncture can ease the symptoms of pain. But acupuncture is also useful in many other non-painful diseases, which has been verified by a lot of random control trials.^[14]^ Acupuncture plays an important role in Traditional Chinese Medicine (TCM) and is commonly used for treating urinary retention in Mainland China. The AUA and GAU guidelines do not report acupuncture for urinary retention.^[1,2]^ TCM believes that the production of urine mainly depends on the bladder qi (Traditional Chinese Medicine believes that qi is a very subtle substance with strong vitality in the human body, and it is one of the basic substances that constitute and maintain human life activities) transformation function, which related to whether the kidney qi is sufficient, and the production of urine is mainly due to the dysfunction of bladder gasification function. According to the meridian theory, acupuncture and moxibustion can cure urinary retention by regulating bladder qi transformation and promoting qi to dredge water passage. Although Traditional Chinese Medicine doctors use acupuncture to treat urinary retention caused by various reasons in clinical practice, there is still a lack of corresponding multicenter randomized controlled trials. Although there are many meta-analysis^[15,16]^ of urinary retention caused by related individual reasons, it is not comprehensive enough, which is not consistent with clinical practice. This study aims to assess the effectiveness and safety of acupuncture for urinary retention.

## 2 Methods

The research has been registered and approved on the PROSPERO website. The registration number is CRD42019119238. Each step of this review strictly refers to the Cochrane Handbook 5.2.

### 2.1 Inclusion/exclusion criteria

The subjects were patients with clinically confirmed urinary retention; studies were randomized controlled clinical trials (RCTs); acupuncture was used in the treatment group, with unrestricted acupoints or manoeuvres, and the control group was treated with non-acupuncture treatment; if the full text is still not available by contacting the author, we will exclude it; the study must have the following outcome measures: primary endpoints are cure rate and post-void residual (PVR); secondary endpoints are overall effectiveness rate, and safety outcome was adverse events.

### 2.2 Study selection

Published literature was retrieved in databases including China National Knowledge Infrastructure (CNKI), PubMed, Web of Science, Cochrane Library, Scopus, EBSCO. Publications available from the inception of databases to June 23, 2020, were reviewed to find the appropriate randomized control trials of acupuncture for urinary retention. The Chinese retrieval words included “Niaozhuliu” or “Longbi” (which means “urinary retention”), “acupuncture”, and “randomization”, and the English retrieval words included “urinary retention”, “retention, urinary”. “acupuncture”, “randomized controlled trial”, “clinical trial”, “RCT”, “random”, and “randomization”. Based on the characteristics of different databases, retrieval strategies including both subject words + free words and keywords + full text were applied. The retrieval strategy is illustrated by the example used for searching PubMed shown in bellow (Table 1).

**Table 1:**
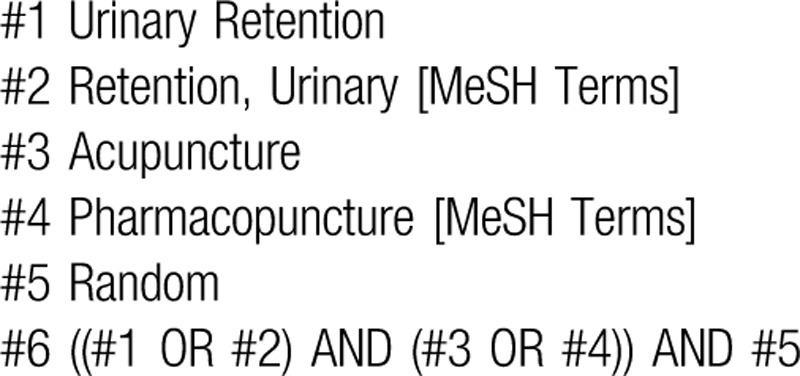
PubMed search strategy.

### 2.3 Study characteristics

The authors searched the database and filtered all references independently (Qinyu Zhao and Bao Song). If the 2 reviewers differed in their decision to include a study, the disagreement was resolved by discussion. If the consensus still cannot be achieved after discussing, we would seek a third party for advising (Hongling Jia and Shenyang Liu). The initially identified articles were imported into Note Express. Initial screening was performed based on inclusion/exclusion criteria and after reading literature titles and abstracts. Full texts were acquired and read for eligible articles or articles that might meet the inclusion criteria to decide whether they would enter the final analysis. When disagreements were associated with either the design of publications or the outcomes of trials such as not reporting safety issue and did not conduct follow up, corresponding authors were contacted to confirm the data that we extracted from their publications or to clarify any ambiguity via email or telephone.

### 2.4 Risk of bias within studies

Two reviewers used Cochrane Handbook for Systematic Reviews of Interventions 5.2 to evaluate the risk of bias in the included literature respectively,^[17]^ which covered the random sequence generation, allocation concealment, blinding, data integrity, selective reporting of positive and/or negative findings, and other sources of bias. One point for each item in the literature. Among them, the “other sources of bias” included the following:

1. whether the experimental design is practical;
2. whether the baseline data is comparable;
3. whether there is a clear conflict of interest leads to an increase in bias;
4. are there clear inclusion and exclusion criteria.

In the “Selective reporting” aspect, due to the lack of registration protocols in include literature, the risk of bias is defined as low if there is a clear efficacy evaluation index. If the 2 reviewers differ in determining the bias in the literature bias, the differences are resolved through discussion. If there is still no consensus after discussion, we will seek advice from a third party (Hongling Jia and Shenyang Liu). Only literature with a score greater than 5 will be included.

### 2.5 Data extraction

We designed a data extraction table, which mainly includes the following aspects:

1. basic information of the studies;
2. research methods and their possible biases;
3. type of urinary of retention;
4. interventions;
5. outcome measures, including PVR, MCC, MFR, BC, and effective rate data;
6. research findings; and
7. adverse events. Data were extracted and verified independently by 2 reviewers.

### 2.6 Statistical analysis

Quantitative analysis was performed by meta-analysis using Cochrane collaboration software RevMan 5.3.5. The relative risk (RR) was selected as the statistic for dichotomous data; the continuous variables are described using mean difference (MD) and 95% confidence interval (CI). During the heterogeneity test, Chi-Squared test was performed firstly; based on its finding, estimates of heterogeneity (*I*^*2*^) were applied. A fixed-effect model was used when *I*^*2*^ was ≤50% and the *P* value was ≥.10; when *I*^*2*^ was >50% or the *P* value was <.10, random-effect model was applied. If the heterogeneity is high, we use Stata 15 for regression analysis to find the source of heterogeneity and then try to use subgroup analysis to increase the stability of the meta-analysis.

### 2.7 Measurements of publication bias

When the same endpoint contains a sufficient number of articles (more than 10 articles) to solve the same problem (more than 10 articles), the funnel chart is used to measure the publication bias.

### 2.8 Paper writing

**Figure 1:**
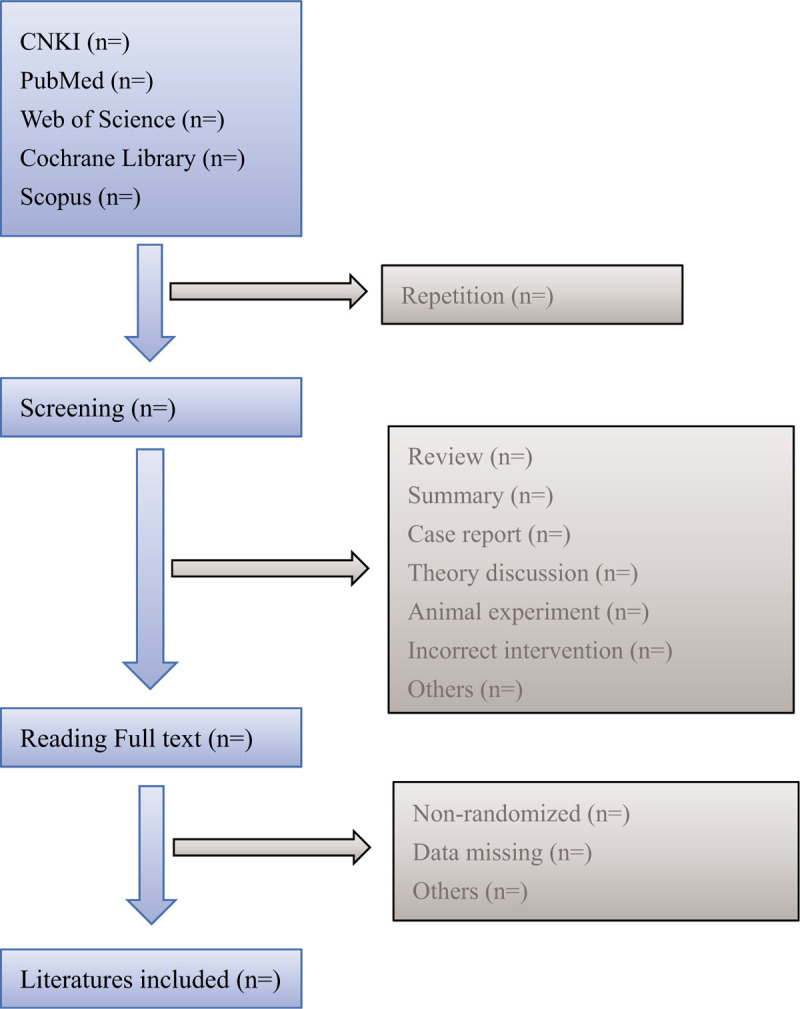
Flow diagram of literature retrieval.

Strictly abide by the PRISMA 2009 checklist requirements during the writing of the paper, and adopt the standard PRISMA 2009 flow diagram (Fig. 1).

## 3 Discussion

UR is a common complication after anesthesia and surgery. Older men are also troubled by UR for a long time. However, catheterization as the most common treatment method when UR occurs often increases the risk of urinary tract infections, and removing the catheter as soon as possible can effectively reduce the risk of infection. Traditional Chinese Medicine doctors have used acupuncture to accelerate the recovery of bladder function for a long time, and have the advantages of simple operation, low price and convenience. However, there is still a lack of corresponding multicenter randomized clinical controlled trials and more comprehensive meta-analysis. This review will be conducted when there is sufficient high-quality literature. To provide convincing evidence and better guide clinical practice, this review will be conducted in strict accordance with the requirements of Cochrane Manual 5.2.

## Author contributions

**Conceptualization:** Qinyu Zhao, Bao Song, Hongling Jia

**Data curation:** Qinyu Zhao, Bao Song, Shenshen Chen

**Investigation:** Qinyu Zhao, Shenshen Chen

**Methodology:** Qinyu Zhao, Bao Song

**Supervision:** Yongzheng Zhu, Hongling Jia

**Validation:** Hongling Jia, Shenyang Liu

**Visualization:** Qinyu Zhao

**Writing – original draft:** Qinyu Zhao, Bao Song, Shenshen Chen

**Writing – review & editing:** Hongling Jia, Shenyang Liu

## References

[1] Oelke M Bachmann A Descazeaud A EAU Guidelines on the treatment and follow-up of non-neurogenic male lower urinary tract symptoms including benign prostatic obstruction. Eur Urol 2013;64:118–40. 23541338 10.1016/j.eururo.2013.03.004

[2] McVary KT Roehrborn CG Avins AL Update on AUA guideline on the management of benign prostatic hyperplasia. J Urology 2011;185:1793–803. 10.1016/j.juro.2011.01.074 21420124

[3] Kaplan SA Wein AJ Staskin DR Urinary retention and post-void residual urine in men: separating truth from tradition. J Urol 2008;180:47–54. 18485378 10.1016/j.juro.2008.03.027

[4] Baldini G Bagry H Aprikian A Postoperative urinary retention: anesthetic and perioperative considerations. Anesthesiology 2009;110:1139–57. 19352147 10.1097/ALN.0b013e31819f7aea

[5] Chung F Recovery pattern and home-readiness after ambulatory surgery. Anesth Analg 1995;80:896–902. 7726431 10.1097/00000539-199505000-00008

[6] Pavlin DJ Rapp SE Polissar NL Factors affecting discharge time in adult outpatients. Anesth Analg 1998;87:816–26. 9768776 10.1097/00000539-199810000-00014

[7] Petros JG Rimm EB Robillard RJ Factors influencing postoperative urinary retention in patients undergoing elective inguinal herniorrhaphy. Am J Surg 1991;161:431–3. 434. 2035761 10.1016/0002-9610(91)91105-r

[8] Keita H Diouf E Tubach F Predictive factors of early postoperative urinary retention in the postanesthesia care unit. Anesth Analg 2005;101:592–6. 16037182 10.1213/01.ANE.0000159165.90094.40

[9] Sullivan NM Sutter VL Mims MM Clinical aspects of bacteremia after manipulation of the genitourinary tract. J Infect Dis 1973;127:49–55. 4683102 10.1093/infdis/127.1.49

[10] Platt R Polk BF Murdock B Mortality associated with nosocomial urinary-tract infection. N Engl J Med 1982;307:637–42. 7110215 10.1056/NEJM198209093071101

[11] Zaouter C Kaneva P Carli F Less urinary tract infection by earlier removal of bladder catheter in surgical patients receiving thoracic epidural analgesia. Region Anesth Pain M 2009;34:542–8. 10.1097/aap.0b013e3181ae9fac 19916208

[12] Yu KW Lin CL Hung CC Effects of electroacupuncture on recent stroke in patients with incomplete bladder emptying: a preliminary study. Clin Interv Aging 2012;7:469–74. 23152677 10.2147/CIA.S37531 PMC3496194

[13] Reston J Now, let me tell you about my appendectomy in Peking. New York Times 1971;1.

[14] Kaptchuk TJ Acupuncture: theory, efficacy, and practice. Ann Intern Med 2002;136:374–83. 11874310 10.7326/0003-4819-136-5-200203050-00010

[15] Wang J Zhai Y Wu J Acupuncture for chronic urinary retention due to spinal cord injury: a systematic review. Evid-Based Compl Alt 2016;2016:1–9. 10.1155/2016/9245186 PMC4846757 27190542

[16] Wang XM Gong J Li SC Acupuncture compared with intramuscular injection of neostigmine for postpartum urinary retention: a systematic review and meta-analysis of randomized controlled trials. Evid Based Complement Alternat Med 2018;2018:2072091. 29861766 10.1155/2018/2072091 PMC5976954

[17] Higgins JPT Thomas J Chandler J Cumpston M Li T Page MJ Welch VA Cochrane Handbook for Systematic Reviews of Interventions version 6.0 (updated July 2019). Cochrane, 2019. Available from www.training.cochrane.org/handbook 10.1002/14651858.ED000142 PMC10284251 31643080

